# Implantation of Teeth and Pericemental Life

**Published:** 1886-10

**Authors:** William J. Younger


ARTICLE III.

### IMPLANTATION OF TEETH AND PERICEMENTAL LIFE.

BY WILLIAM J. YOUNGER, M. D.

[Read before the California State Dental Association.]

*Mr. President and Gentlemen:*—

Dr. Warner, Chairman of the Committee on Surgery and Pathology, did me the honor to say, to-day, that the only new thing in Dental Surgery, that he was aware of, was the successful transplantation of teeth into artifical sockets by Dr. Younger, one of the Committee; several of which operations had been witnessed by himself, and the success of which he could vouch for. He, therefore, delegated to Dr. Younger the task of making out the report of the Committee. Had Dr. Warner informed me of this intention sooner, I should have been able to present to you a report fuller in detail and more free from crudities than this, of necessity, must be.

Transplantation and Implantation, as I call my new operation, has so far realized my most sanguine expectations, and leads me to the belief that in a short while it will become as firmly established in professional practice as any other operation requiring skill and judgment.

Since the publication of my brochure on "Transplantation of Teeth into Natural and Artificial Sockets," which you have all seen, and a portion of which formed a report made to this Association, at its last session, I have made a discovery in regard to the vitality of the pericementum, that is even more startling than the success of the operation of Implantation itself. In the pamphlet I recommended the use of cock's combs as a means of preserving the vitality of the peridental membrance, and also mentioned that in two instances the life of this membrance had been preserved for over fifty hours, in tepid water. I now, however, have to report a case—one of several—which proves that these means are not at all necessary to preserve the vitality of this, the most wonderful tissue in the human body; that this vitality of the pericementum is marvelous; and that it may be as tenacious as that inherent in the seeds of plants.

In the early part of March, 1886, Mrs. Dr. H. G. Blankman—the wife of one of the pioneer dentists of this coast—brought me a bicuspid that had been extracted at her solicitation, in Sacramento, on the 31st day of January, 1885, in the belief that it was the seat of a neuralgic pain, which had been the cause of great anguish to her. This tooth, brought to me after this long lapse of time, had in the meanwhile been carried about in her portemonnaie, stowed away in her jewel case and shuffled about in her bureau drawer. And this tooth she wanted replanted in her jaw! My first impulse was to laugh, my next to argue with her about the impossibility of success of such an operation, explaining to her that it was due only to the vitality of the membrane covering of the root that the operation owed its success; that, without this living membrane, the tooth was as impossible of attachment as so much bare ivory or porcelain; and that while I had succeeded in keeping this membrane alive for over two days, it was by constant immersion in warm water, at a blood temperature; but that the pericementum of this tooth was, as she herself could see, as dry and shriveled as parchment, and as devoid of life.

Just as I had persuaded her of the impossibility of success, there flashed through my mind a passage in John Bell's work on the "Anatomy and Physiology of the Human Body," that I had lately read, which awoke the suggestion that success in implanting that tooth was, after all, possible.

This eminent surgeon, in criticising an article of the famous John Hunter, says: "How can such vitality exist independently of a circulation? But there are not wanting examples of an obscure and low degree of life existing in animals' ova, or seeds, for seasons without a circulation; and if for seasons, why not for a term of life?"

While this passage did not bear directly on the subject in question, it somehow awoke a train of thought that led me to the conclusion, that in that dry, shriveled membrane there possibly lay lurking a dormant life, which under favorable conditions would rouse its energies and make the tooth enclosed once more a living, useful organ.

I had proved that the peridental membrane possesses a wonderful tenacity of life, in at least two instances, where after it had been removed from all life-giving connections for fifty-two hours, it was as vigorous in forming attachments as though it had been planted immediately after removal. I therefore reasoned with myself, if this peridental membrane preserves a vitality unimpaired for fifty-two hours, why not for so many weeks or months? So I said: "Mrs. Blankman, the idea has just occurred to me that what you want done is, perhaps, possible." And I explained the cause of this revolution of opinion, as she is not only a brave but a very intelligent woman; and continuing, said: "I will perform this operation as an experiment, to test the vitality of the pericementum, for, though I have no positive expectation, I have a hope of its success." So, on the 11th of last March, in the presence of and with the assistance of Dr. Alexander Warner, who was acquainted with all the circumstances of the case, I drilled a socket between the first left superior bicuspid and first molar; and after soaking the tooth in water—temperature 120° Fah.—for twenty-five minutes, to soften the membrane, restored to the jaw that which it had been deprived of just thirteen months and eleven days before.

As the dental aspect of the tooth was perfect, and the approximal not nearly so much so, I turned the tooth, thereby much improving the original appearance of that portion of the mouth. When the operation was finished, the tooth was found so firmly fixed in the socket that retaining ligatures were not applied; and union took place as rapidly and as thoroughly as if it had been a fresh tooth.

The tooth retaining this firmness and no swelling nor pain ensuing, the lady commenced eating with it, and at the end of twelve days became so careless in her use of the tooth, that she bit a hard crust of French bread with it. This was too much; the tooth received a wrench that loosened it and caused the gum to bleed profusely. Next morning she hurried to the office, and with tears in her eyes, narrated the accident. I found the tooth quite loose, but not dropping, and the gum on the palatine surface swollen, and with the evidence of having bled at the margin.

In my heart I was glad the accident had happened; for to me it was a test of the question whether the retention of the tooth was due simply to the nice adaptation of the walls of the socket to the root of the tooth, and therefore only mechanical, or whether it was really due to awakened life in the peridental membrane, and consequent vital connectiou with the living environment of gum and alveolar substance.

If mechanical, I argued, the irritation that has been set up around it, especially in its present loose condition, will cause its expulsion; but if vital, it will be retained and become firm again. I therefore did not seek to retain it in place by any ligature, but simply painted the gum with tincture of iodine, and cautioned her not to chew on that side until I gave her permission. In one week all marks of the accident had passed away; the tooth became again firmly fixed, and remains to the present moment as solid as a rock. I have tried, since then, to pass a delicate instrument, the point of which had been flattened for the purpose, between the gum and the tooth; but the act gave as much pain, and the instrument met with as much resistance, as in the tissues surrounding the teeth that had never been disturbed in their sockets; all of which clearly proves that the peri cementum of that tooth—dry and shriveled as it was—had during those long months of absence from any life-supporting substance, tossed about from place to place, from pocket and purse to casket and drawer, preserved a vitality as fresh and vigorous as when it was removed from the place in which it grew.

I am happy to say that Mrs. Blankman has kindly consented, in the interest of science, to present herself to you this afternoon, in order that you may examine this tooth, and satisfy yourselves by personal and thorough examination, as to the success of the operation. And I want each and every one of you gentlemen to test in every way that your ingenuity may suggest—short of extraction —the statement I have made, that vital connection has been established between that tooth and the walls of the socket, as perfect as that of the other teeth that have grown there and never been tampered with.

I have since tried implanting teeth which have been extracted for weeks and months, with equal success, proving the wonderful tenacity of life in the peridental membrane.

The question now in my mind is: "When does the pericementum die?"

In consequence of these experiments, and the equal success of the implantation of long extracted teeth with that of fresh ones, I have discarded, as unnecessary, the warm water and the comb of the troublesome cock.

I now simply lay the teeth aside in a clean, cool, dry place, and prepare and use them as I want them.

I have also discarded the flat drill, and use, instead, graded trephines for piercing the bone and for doing the major part of the work; finishing the walls, as formerly, with burs of various shapes.

Much doubt has been expressed by the profession in the East and elsewhere as to the stability of implanted teeth, because the kindred operations of Replantation and Transplantation that have come under their observation, and which, at first, gave promise of permanency, have so generally turned out failures in one or two years, from absorption of the roots. They argue that if Replantation, which is the putting back of a tooth into the socket from which it has been but just drawn; and Transplantation, which is the planting of a stranger tooth into a socket from which its own has been but freshly extracted; if, they say, these cognate operations, where the conditions seem so much more favorable, in consequence of the sockets being natural, are so generally failures, what better result can be expected from an operation where the socket is formed by violence to the jaw?

Now, if they would but consider the conditions and circumstances under which these different operations are undertaken, they would readily see that the premises upon which they ground their assumption are unsound and deceitful. For instance, in replantation, as this operation is usually performed, the parts—that is, the peridental membrane, the apex of the root and the alveolar process immediately surrounding these—are highly inflamed, in a state of disease, with pus either already formed or forming at the end of the root, and the operation is undertaken with the view of relieving or aborting an alveolar abscess. A portion of the diseased apex is then cut off, and the tooth is forced back into the cavity. Here we have a diseased root thrust back into a diseased socket. The disease is not remoued, its conditions are simply modified; and while the congestion may subside, and the tooth become comparatively comfortable, the disintegration of the root substance—already begun—is likely to continue, and in the course of time the entire root becomes destroyed, or, what is called absorbed; and the bodiless crown drops off. So much for Replantation.

Again, in Transplantation there is a healthy tooth, but it is usually made to take the place of a miserable, old, diseased root, that has been growling and festering in a diseased socket for years, to the discomfort of its unfortunate possessor. The diseased root is pulled out, but is the disease in the surrounding alveolus extracted with it? On the contrary, enough is usually left in its tissue to make war upon the new occupant, and either cause its expulsion, or eat away its substance. You must remember, that while the old root remained, there was sufficient vent through its decayed or broken structure to permit the gases of decomposition and the pus to escape, and thus prevent active trouble. But when the new tooth is put in, the vent is entirely occluded; and if there be sufficient disease in the alveolus, the retained gases and pus effect the expulsion or the painful elongation of the intruder. If not enough disease is left to do this, then the slow process of erosion is apt to ensue, and the root becomes, in time, absorbed.

Now, in *Implantation we have a healthy root in a healthy socket*; and therefore, the factors that tend to the destruction of the root in Replantation and Transplantation are not present, and, therefore, not operative in Implantation.

As to the seeming violence to the bony structure of the jaw, I will state that there is no substance in the human body that seems so tolerant of abuse as this same alveolar process. And my experience is, that union takes place more readily and kindly, and the teeth become much sooner firm and serviceable in Implantation than in either of the other operations.

I trust, my brother practitioners, that what I have read to you will serve to dispel whatever gloomy forebodings you may also have entertained, in considering the future of Implantation, and that you will apply yourselves at once to master this operation, for the benefit of your patients and of yourselves. Also, I urge on you the practice of Replantation and Transplantation, for they can be made uniformly successful, if you but follow the system I have made you acquainted with, and the primal rule of which is, to allow no disease to remain in socket or in root. Do not wait half a lifetime, as the pessimists in the profession would have you do, in order to find out if the operation is going to be successful, but commence now.

In this paper I have summarized these operations as performed by our Eastern brethren, in order to make their argument as effective as possible, and so to impress upon you the reason of the failure of Replantation and Transplantation, as practiced by them, and to demonstrate that to the disease left in the socket and root, or in the socket alone, must be attributed the failures caused by absorption of the root; otherwise, why should the result of my experience in these operations be so different from theirs? In all my practice of these kindred operations—and it has probably far exceeded that of any other practitioner in the United States—I have had but one case of non-success, due to the absorption of the root. As it has a lesson in point, I will narrate it.

In this case, the tooth inserted was but poorly covered with pericementum, and the socket had been diseased for eighteen years, and was, moreover, so much larger than the root that the tooth had to be held in by ligatures. The evening after its insertion, the ligature loosened and the tooth fell out and dropped on the carpet. The gentleman placed it immediately back in the socket, and being unable either to tie it or find me, retained it in a position by closing his jaw and keeping his teeth tightly pressed together by means of a handkerchief passed under his chin and tied over his head. At nine o'clock the next morning I found that attachment had already taken place, which assured me that it had not been fatally injured by the episode of the night previous. I therefore renewed the ligatures, and when the gentleman started for his English home, two months later, the tooth was well attached, though not firm. This operation was performed March 2d, 1885; and a letter, dated more than a year later, informed me that the crown had broken off, and he was puzzled to know why it should have done so, when he was not cracking nuts with it! He also wrote that the fangs remained imbedded; but in this he must be mistaken. It is, in my mind, a case of absorption of the roots.

It is the only case where I did not either cut out the disease nor treat it sufficiently long to satisfy myself that it was all gone from the socket; and it is the only tooth I have lost by absorption. I may say that in cases like this, where the size of the socket is in excess of the diameter of the root, either from natural causes or alveolar disease, I form an artificial root of gum shellac, of the shape and a trifle smaller than the body of the root to be inserted; to which I attach an artificial crown. This is inserted into the socket in the direction the new tooth is to occupy, and there given temporary lodgment; by which means the margins of the gums and alveolus are kept from retracting, the socket made healthy, and the granulations allowed to fill the cavity, until there shall be perfect contact with the whole surface of the new tooth, which I then put in.

